# An Adaptive OFDMA-Based MAC Protocol for Underwater Acoustic Wireless Sensor Networks

**DOI:** 10.3390/s120708782

**Published:** 2012-06-27

**Authors:** Issa M. Khalil, Yasser Gadallah, Mohammad Hayajneh, Abdallah Khreishah

**Affiliations:** 1 College of Information Technology, United Arab Emirates University, Al Ain 17555, United Arab Emirates; E-Mail: mhayajneh@uaeu.ac.ae; 2 Department of Electronics and Communications Engineering, Misr International University, Cairo 11311, Egypt; E-Mail: ygadallah@ieee.org; 3 Department of Electrical and Computer Engineering, New Jersey Institute of Technology, Newark, NJ 07102, USA; E-Mail: akhreish@gmail.com

**Keywords:** MAC protocols, sensor networks, OFDMA, CDMA, channel access/reservation

## Abstract

Underwater acoustic wireless sensor networks (UAWSNs) have many applications across various civilian and military domains. However, they suffer from the limited available bandwidth of acoustic signals and harsh underwater conditions. In this work, we present an Orthogonal Frequency Division Multiple Access (OFDMA)-based Media Access Control (MAC) protocol that is configurable to suit the operating requirements of the underwater sensor network. The protocol has three modes of operation, namely random, equal opportunity and energy-conscious modes of operation. Our MAC design approach exploits the multi-path characteristics of a fading acoustic channel to convert it into parallel independent acoustic sub-channels that undergo flat fading. Communication between node pairs within the network is done using subsets of these sub-channels, depending on the configurations of the active mode of operation. Thus, the available limited bandwidth gets fully utilized while completely avoiding interference. We derive the mathematical model for optimal power loading and subcarrier selection, which is used as basis for all modes of operation of the protocol. We also conduct many simulation experiments to evaluate and compare our protocol with other Code Division Multiple Access (CDMA)-based MAC protocols.

## 1. Introduction

Underwater acoustic sensor networks (UWASNs) are used in many applications where accurate and timely phenomena detection is required. Application examples include tsunami warning, pollution monitoring, coastal protection, communication among divers, and surveying the ocean floor in search of new resources. UWASNs can be deployed in self-configurable random arrangements where network nodes cooperate to capture and disseminate data.

It is a known fact that electromagnetic waves cannot propagate for long distances in seawater [1]. Therefore, acoustics provide the most obvious choice to enable underwater communications. Acoustics, however, have had limited success in shallow water, despite being used effectively for point-to-point communication channels in vertical deep water. In shallow water, communication channels exhibit many imperfections due to many factors such as shipping noise, multipath transmission, water motion, density gradients, and the non-homogeneity of the water due to particles of solid or gaseous matter. Of these factors, there are two major elements that limit acoustic communications in shallow water; namely, time-varying multi-path propagation and colored background Gaussian noise. In shallow water, propagation occurs in surface-bottom bounces in addition to a possible direct path. Channel characteristics vary with time due to random signal fluctuations which include surface scattering due to waves, which is the most important contributor to the overall time variability of the shallow water channel. The combination of the previous factors results in a time-varying multi-path propagation pattern. This in turn increases the inter-symbol interference and causes frequency dependent fading, thus limiting the communication data rates. Although adding adaptive channel equalization filters can help reduce the problem, with carrier signals falling below certain noise no equalization can rectify the problem.

In this work, we propose an adaptive MAC protocol for underwater sensor networks. The protocol is configurable to suit the operating conditions of the UWASN. Our protocol is based on Orthogonal Frequency Division Multiple Access (OFDMA). OFDMA is a special case of Multi Carrier Modulation (MCM) in which multiple user symbols are transmitted in parallel using different subcarriers with overlapping frequency bands that are mutually orthogonal. It is the most promising technology that can deliver a wireless acoustic signal much farther with much less inter-cell interference (note that intra-cell interference is zero) than competing technologies due to the orthogonality of the subcarriers. The orthogonality among these subcarriers also enables the most efficient use of the scarce acoustic spectrum. OFDMA converts a selective fading acoustic channel into parallel independent acoustic sub-channels that undergo flat fading. Multipath delay spread of the acoustic channel that causes Inter-Symbol Interference (ISI) is handled completely by using the Cyclic Prefix (CP). Compared to other technologies, OFDMA is the most efficient in exploiting *multiuser diversity* (*i.e.*, different nodes experience different channel fluctuations) in wireless multipath fading channels. Due to multiuser diversity and frequency diversity, OFDMA may perform better in shallow water than in deep water, in comparison to other technologies. These features also allow simultaneous activities between neighboring nodes while avoiding collisions. This is done through the careful assignment of subcarriers to neighboring nodes. This powerful feature of our MAC protocol eradicates the hidden terminal and the exposed terminal problems that are encountered with most of the other UWASN MAC protocols. The adaptive nature of proposed algorithm affects the way the subcarriers are assigned to network nodes. For example, when the equitable mode of operation is enabled, the subcarriers are divided equally among the nodes in an optimal manner according to the selection model that we derive in this study. This work extends our previous work in [2] by providing more accurate underwater model that better captures the unique characteristics of acoustic waves in water. Additionally, this work proposes, analyses and compares three different modes of operation for the proposed protocol compared to the single mode of operation in [2]. Finally, this work provides extensive simulation experiments that evaluate a wider range of parameters which enables us to draw better insights and conclusions about the proposed protocol.

Previous studies [3,4] suggested the use of *single-user* OFDM modulation at the physical layer for the design of underwater acoustic OFDM modems. To the best of our knowledge, this study is the first to exploit the features of OFDMA to build an OFDMA-based UWASN MAC protocol that enhances *multi-access* in bandwidth-limited imperfect underwater acoustic channels.

The remainder of this paper is organized as follows: in Section 2, we present the related work. In Section 3, we present system and channel models. In Section 4, we derive the mathematical model for optimal subcarrier selection. In Section 5, we describe the proposed protocol and present its various modes of operation. In Section 6, we present the experimental results. Finally, we conclude the paper in Section 7.

## 2. Related Work

There has been intensive research on MAC protocols for terrestrial wireless sensor [5] and *ad hoc* networks [6]. However, due to the different nature of the underwater environment and applications, there are several issues related to the suitability of terrestrial MAC solutions in the underwater environment. For example, channel access control in underwater acoustic sensor networks poses challenges such as the limited bandwidth, very high and variable propagation delay, channel asymmetry, and heavy multipath and fading phenomena.

There have been several studies that have discussed and derived MAC protocols for UWASNs. In [7], the authors summarize some of the MAC protocols that have been proposed for UWASNs. The multiple access control including Frequency Division Multiple Access (FDMA), Time Division Multiple Access (TDMA), Code Division Multiple Access (CDMA) and the medium access control including ALOHA, Carrier Sense Multiple Access (CSMA), Multiple Access with Collision Avoidance (MACA), and Floor Acquisition Multiple Access (FAMA), which use in terrestrial sensor network, have been modified in order to adapt to the nature of acoustic transmission in an underwater environment.

Slotted FAMA [8] uses time slotting over floor acquisition multiple access (FAMA) discipline where the time line is divided into fixed-length slots. It combines both carrier sensing (CS) and a dialogue between the source and destination prior to data transmission. Although time slotting eliminates the asynchronous nature of the protocol and the need for excessively long control packets, guard times should be used in the slot duration. Moreover, Slotted FAMA assumes an infinite local buffer at each node which is unrealistic in resource limited sensor nodes. Finally, Slotted FAMA assumes that the clocks of the sensor nodes are fully synchronized.

In [9,10], the authors use contention instead of Request To Send/Clear To Send (RTS/CTS) packets to acquire the channel in a distributive manner. This algorithm, namely T-Lohi, divides time into slots, but uses tones instead of control packet exchanges to contend for the channel. However, T-Lohi ignores the hidden terminal and the exposed terminal problems. Furthermore, the use of tones requires the use special hardware which increases the cost and the complexity of the sensor nodes.

In [11], the authors propose a MAC protocol for dense networks. The used performance metric is energy consumption rather than the bandwidth. The algorithm is designed to achieve a locally synchronized schedule even in the presence of long propagation delays. The authors assume synchronized node clocks. However, it is not clear how nodes go about reselecting transmission cycles when there is an overlap between two nodes. Moreover, the strategy for resolving hidden terminal problem was not clarified. In addition, having a specific time for data transmission even when there is no data to send causes delays for other nodes having data to send.

The study in [12] introduces a MAC protocol for non-synchronized energy-constrained UAWSN which are characterized by long propagation delays. The proposed protocol takes advantage of the greater received power over short links to reduce their handshake length. Therefore, a handshake only needs to avoid collisions from nodes closer than a certain distance. Hence, handshakes between close neighbors can be made shorter but those between far-apart nodes need to become increasingly longer. However, the RTS/CTS mechanism that is used to solve the hidden terminal problem incurs additional delay to avoid collision while transmitting data.

In [13] the performance of Aloha-based protocols in underwater networks is studied and two schemes are proposed. The schemes, namely, Aloha with Collision Avoidance (Aloha-CA), and Aloha with Advance Notification (Aloha-AN), are capable of using the long propagation delays to their advantage. However, the protocols do not address the problem of hidden and exposed nodes. The limitation of ALOHA protocols in underwater environments was analyzed in [14,15]. In [14], the results show that long propagation delay of acoustic signals using the ALOHA technique prohibits the coordination among nodes thus resulting in no performance gain. Although the nodes send messages in pre-defined time slots, there is no guarantee that they will arrive in these time slots. The simple analysis and simulation results show that Slotted ALOHA exhibits the same utilization as non-Slotted ALOHA. Moreover, in [15], the authors identify the challenges of modeling contention-based medium access control protocols and present a model for analyzing ALOHA variants for a simple string topology as a first step toward analyzing the performance of contention-based proposals in multi-hop underwater acoustic sensor networks. Collisions are the most limiting factor in the performance of the different ALOHA variants.

Several Multiple Access Collision Avoidance (MACA-based) protocols have also been proposed in [16–19]. In [16] the authors propose an asynchronous random access MAC protocol; namely, MACA-MN (short for MACA in Multi-hop Networks). The protocol utilizes a handshaking-based approach in order to help avoid collisions and alleviate the hidden terminal problem in multi-hop underwater networks. In addition, the MACA-MN can overcome the low throughput problem suffered by typical handshaking-based protocols (such as MACA), by transmitting a train of packets during each round of handshake. The enhancement made in [16] is in the simultaneous formation of the packet train for multiple neighboring nodes and in relaxing the synchronization requirement. The work in [17] presents an asynchronous random access MAC called the MACA-DT (short for MACA for Delay Tolerant) protocol. The protocol utilizes a typical MACA handshaking-based approach in addition to using adaptive silent time and simultaneous handshake technique to overcome the low throughput and the long end-to-end delay problems of typical handshaking-based protocols. In [18], a protocol that provides methods to handle two types of data namely, burst data and instant messages is proposed. In the burst data mode of operation, a connection establishment is made via a handshaking technique while this is not needed in case of the instant messages mode. In [19], the authors propose a protocol dubbed Geographical MACA (G-MACA). It assumes that nodes know their locations and those of their neighbors by exchanging beacons periodically. This, in addition to the regular MACA RTS/CTS exchange places a burden on the limited bandwidth. The main issue with MACA based protocols, in general, is the demand on bandwidth in the handshaking process which overburdens the already scarce bandwidth resources.

Adaptive Propagation-delay-tolerant Collision Avoidance Protocol (APCAP) [20] is a collision avoidance MAC that employs an improved handshaking mechanism using RTS and CTS frames. However, [20] mainly considers throughput and energy efficiency and does not consider the unique underwater acoustic environment issues such as fading and long and variable propagation delay.

Several CDMA-based techniques have been proposed, [21–23]. In [21] a distributed MAC protocol called UW-MAC is described. UW-MAC is a transmitter-based Code Division Multiple Access (CDMA) scheme. UW-MAC aims at guaranteeing high network throughput with low channel access delay, and low energy consumption. However, UW-MAC lacks a clear mechanism that addresses the hidden and exposed terminal problems. Moreover, it requires that all nodes have knowledge of all other nodes' Multiple Access Interference (MAI), which may be prohibitive in terms of capacity of low capability sensor nodes. In [21], the authors present a tree-based technique where a periodic sleeping mode is employed for the purpose of saving energy. A hierarchical tree-topology is considered where a staggered-wakeup pattern is adopted to enable a receiver node to listen just at the time when a long-delayed packet arrives. The sensor nodes located in same hierarchical level are multiplexed by means of different orthogonal codes. In [23], the authors propose a technique that depends on ordering nodes within a path to the sink node according to their positions within this path. A node in the path is not allowed to send a packet that it has until it receives a packet from the previous node on the path. This previous node cannot delete this packet it sent until it receives it back from the subsequent node on the path. To avoid collisions at the sink, each path is assigned a different CDMA spreading sequence. The algorithm has several issues. It assumes full knowledge of each routing path, which is not the role of a MAC protocol. It imposes high delays due to the need to wait for packets from previous nodes on the path before a node can send its own. It also requires unnecessary retransmissions which wastes the already tight bandwidth.

In [24], a Carrier Sense Multiple Access (CSMA-based) MAC protocol named Propagation Delay Aware Protocol (PDAP) is proposed. This protocol aims at maximizing the bandwidth utilization while enabling interleaved and reliable communications between different pairs of nodes. However, in multi-hop transmissions, interfering nodes may compromise successful packet reception. Also, due to lack of information of all on-going communications in remote parts of the network, PDAP nodes cannot reliably acquire the channel.

In [25], A MAC protocol that segregates the available bandwidth into a small control channel and a data channel that occupies the majority of the bandwidth is proposed. Reservations for main channel time are made by transmitting RTS packets on the control channel. The aim of this protocol is to achieve a better channel utilization for a given data bandwidth. However, this protocol does not consider channel variations due to fading.

The work described in [26,27] depends on determining specific time slots for different network nodes. In [26], the authors propose a TDMA-based approach for their MAC protocol. The authors assume that the distance between the sensor nodes and the sink is known, without specifying the method, and they use this as the basis for calculating the timing schedule, which is an unrealistic assumption. Also, the exchange used for synching the nodes consumes considerable bandwidth, which is one of the important drawbacks in using TDMA in underwater environments where the BW is limited. Somewhat similar to this approach, the study in [27] which depends on reserving specific time slots to enable network nodes to transmit during their particular time slots. It depends on synchronizing all network nodes, which is a main disadvantage of the protocol.

Several OFDMA based techniques have been proposed such as [28] and [29]. In [28], the authors propose a UW-OFDMAC protocol that aims at setting the optimal combination of the transmit power, subcarrier spacing and guard interval duration at the transmitter side so as to minimize the energy consumption and mitigate the ICI and ISI effects. It assigns mutually exclusive sub-channels to different users. In [29], the authors introduce a technique in which the sink node allocates OFDMA sub-channels to sensor nodes based on their distance from the sink node. The distance is measured through the use of round trip times, which assumes that network nodes must be kept in full synchronization. It is not clear how collision will be dealt with as the frequency bands are allocated to groups of sensors that have similar distances from the sink node. The technique also relies on the exchange of considerable control traffic messages, which casts doubts about its applicability in the bandwidth-limited underwater environment.

In [30], the authors propose a protocol that is based on one mobile node that moves close to the other network stationary nodes to collect their data in a single hop fashion. The mobile node signals its presence in the vicinity of a certain node by using a beacon. The protocol relies on the use of scheduling in order to avoid collisions. Again similar to [29], this implies the need to have all network nodes synchronized. It is obvious that the use of this protocol requires specific network conditions such as the presence of a mobile node that is needed to perform data collection.

In [31], the authors propose a multichannel technique that is based on the cyclic quorum concept. This technique is targeted for heavily loaded high contention networks. The authors assume the existence of m equal-bandwidth channels and the full synchronization of network nodes. These assumptions severely limit the usefulness of this technique as it can only be used in network with special node capabilities.

## 3. System and Channel Models

### 3.1. System Model

**Figure 1.**
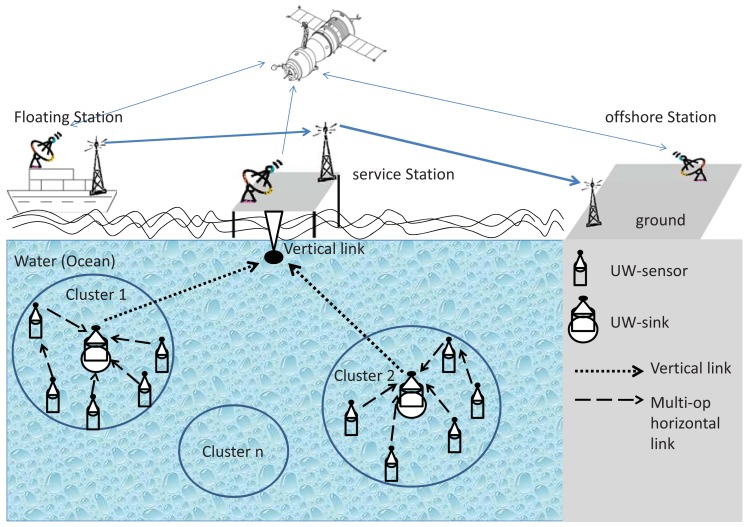
Underwater acoustic sensor network architecture.

We assume that the sensor nodes are deployed vertically under the sea surface in a 3D display, typically anchored to the sea bottom (Figure 1). Therefore, the network nodes are located at different depths or horizontal planes in shallow water. Sensor nodes may be clustered around a sink node (UW-sink in Figure 1) that is capable of aggregating the data collected by the sensors (UW-sensors in Figure 1). The sink node communicates the collected data to a processing center that is either located under the sea surface (e.g., a submarine), a surface ship, or a command center which also can send reprogramming commands to UW-sensors through UW-sinks [32,33]. The sink node acts, in addition to its role within the sensor network, as a gateway to external endpoints or networks. In this kind of network deployments, it is conceivable to expect either multi-hop or single-hop communications between each sensor node and the sink. The exact communication pattern (single-hop, multi-hop, or hybrid) depends on the area that needs to be covered as well as the strategy of the deployment. In this work, we address the most general case of multi-hop communication between the sensor nodes and the sink. We also assume a heterogeneous network structure; *i.e.*, a few sensor nodes of limited capabilities and one or more highly capable sink nodes from both computational and communication standpoints. We consider a fully distributed architecture to be able to develop a technique that works with the general deployment scenario of such networks.

### 3.2. Acoustic Channel Model

Path loss in underwater acoustic channels depends on both, the distance and the operating frequency between the transmitter and receiver nodes. This is unlike the terrestrial Radio frequency (RF) wireless channels, where path loss only depends on the distance between communicating nodes [34]. According to Urick's model [35], the total path loss that an acoustic signal undergoes for a distance *d* between two communicating nodes at frequency *f* (kHz) dubbed as *A(d,f)* is given by:

(1) $$A\left(d,f\right)=d^{k}\cdot {\left(a\left(f\right)\right)}^{d/1000}$$

where *k* is the spreading coefficient that models the geometry of the acoustic signal propagation. A typical practical value that has been used in the literature of *k* is 1.5. The absorption loss factor, *a(f)*, in Equation (1) models the conversion of acoustic pressure into heat. With *f* expressed in kHz, this factor is approximated by Thorps formula [35] as follows:

(2) $$\begin{array}{ll} A\left(f\right) & =10\log\left(a\left(f\right)\right)\;\text{dB}/\text{km}\\ & =\frac{0.11f^{2}}{1+f^{2}}+\frac{40f^{2}}{4100+f^{2}}+2.75\times 10^{-4}\;f^{2}+0.003 \end{array}$$

As for the noise, four main contributors to the ambient noise have been identified [36], namely thermal noise *N_th_(f)*, turbulence *N_t_(t)*, shipping and human activities *N_s_(f)*, and wind-driven waves *N_w_(f)*. These four sources are modeled by colored Gaussian statistics and continuous Power Spectral Density (PSD). Empirically, the PSD formulae of the four noise sources as a function of frequency in kHz are given by [37] as follows:

(3) $$\begin{array}{l} 10\log\left(N_{t}\left(f\right)\right)=17-30\log\left(f\right)\\ 10\log\left(N_{s}\left(f\right)\right)=40+20\left(s-0.5\right)+26\log\left(f\right)-60\log\left(f+0.03\right)\\ 10\log\left(N_{w}\left(f\right)\right)=50+7.5w^{1/2}+20\log\left(f\right)-40\log\left(f+0.4\right)\\ 10\log\left(N_{th}\left(f\right)\right)=-15+20\log\left(f\right) \end{array}$$

where *s* ∈ [0,1], is the shipping activity factor whose value is close to 0 for low activity and close to 1 for high shipping activity and *w* is the wind speed in m/s, which is used to capture the surface motion, caused by wind-driven waves.

The total ambient noise *N(f)* is given as the superposition of the four noise sources as follows:

(4) $$N\left(f\right)=N_{t}\left(f\right)+N_{s}\left(f\right)+N_{w}\left(f\right)+N_{th}\left(f\right)$$

The signal-to-noise ratio (SNR) of an incoming acoustic tone of frequency *f_i_*, transmitted from node *j* to node *n* with distance *d_jn_* can be expressed as in [36]:

(5) $$\text{SNR}\left(d_{j,n},f_{i}\right)=\frac{{\left|{\alpha_{j,n}}^{i}\right|}^{2}{P_{j,n}}^{i}}{A(d_{j,n},f_{i})N(f_{i})B_{S}}$$

where $P_{j,n}^{i}$ is the transmit power on subcarrier *i* from node *j* to node *n* and *N(f_i_)* is the noise power spectral density given by Equation (4), *B_s_* is the noise bandwidth at the receiver. The small-scale fading due to multipath experienced by subcarrier *i* in the underwater channel between node *j* and node *n* is captured by $\alpha_{j,n}^{i}$. In this paper, we assume that $\left|\alpha_{j,n}^{i}\right|$ follow a Rayleigh distribution as in [38].

## 4. Optimal Subcarrier Selection Model

We base the proposed protocol on a mathematical model that presents the minimum possible transmit power to achieve the required data rate per connection. The transmission on each subcarrier (sub-channel) is assumed to undergo a Rayleigh flat fading variations with background Additive Colored Gaussian Noise (AGN).

Let $P_{j,n}^{i}$ be the power used for packet transmission from node *j* to *n* using subcarrier *i*. The objective is therefore to find the minimum possible transmit power over a group of *x* subcarriers to achieve the required transmission rate, denoted by *R_o_*, between the nodes *j* and *n*. Mathematically speaking, the problem can be formulated as follows:

(6) $$\min\left\{\sum_{i=1}^{x}P_{j,n}^{i}\right\},\;\text{S.t.}\;B_{s}\sum_{i=1}^{x}\log_{2}\;\left[1+\text{SNR}\left(d_{j,n},f_{i}\right)\right]\geq R_{0},\;\text{and}\;P_{j,n}^{i}\geq 0\forall i,j,n$$

where *SNR*(*d_j,n_, f_i_*) is given by Equation (5), and *x* is the number of used subcarriers on which node *j* has selected to transmit data to node *n*. The subcarrier width is denoted by *B_S_* and is given by *B_S_* = *B_T_*/*N*, where *B_T_* and *N* are the total acoustic channel bandwidth (in Hz) and total number of data subcarriers, respectively.

Note that the objective function is convex, also $B_{S}\;\sum_{i=1}^{x}\log_{2}\;\left[1+\text{SNR}\left(d_{j,n},f_{i}\right)\right]$ is a concave function in terms of $P_{j,n}^{i}$. Therefore, the constraints' set is convex. Hence we have a convex optimization problem [39] which makes solving the problem in Equation (6) equivalent to minimizing the following cost function:

(7) $$L\left\{{\left(P_{j,n}^{i}\right)}_{i=1}^{x},\lambda \right\}=\sum_{i=1}^{x}P_{j,n}^{i}-\lambda \left\{B_{s}\sum_{i=1}^{x}\log_{2}\;\left[1+\frac{{\left|\alpha_{j,n}^{i}\right|}^{2}P_{j,n}^{i}}{A(d_{j,n},f_{i})N(f_{i})B_{s}}\right]-R_{0}\right\}$$

where *λ* is the Lagrange multiplier. Taking the derivative, we get:

(8) $$\frac{\delta L}{\delta P_{j,n}^{m}}=1-\frac{\lambda }{\ln2}\left[\left\{\overset{{\left|\alpha_{j,n}^{m}\right|}^{2}}{\;}/\underset{A(d_{j,n},f_{m})N(f_{m})}{\;}\right\}/\left\{1+\left(\overset{{\left|\alpha_{j,n}^{m}\right|}^{2}P_{j,n}^{m}}{\;}/\underset{A(d_{j,n},f_{m})N(f_{m})B_{s}}{\;}\right)\right\}\right]=0$$

Solving Equation (8) for $P_{j,n}^{m}$, we get:

(9) $$P_{j,n}^{m}=B_{s}\;{\left[\frac{\lambda }{\ln2}-\frac{A(d_{j,n},f_{m})N(f_{m})}{{\left|\alpha_{j,n}^{m}\right|}^{2}}\right]}^{+},\forall m=1,2,\cdots ,x$$

where []^+^ here means a projection on the positive real numbers. To obtain *λ*, we use Equation (7) again as follows:

(10) $$\frac{\delta L}{\delta \lambda }=0\Rightarrow B_{s}\sum_{i=1}^{x}\log_{2}\;\left[1+\frac{{\left|\alpha_{j,n}^{i}\right|}^{2}P_{j,n}^{i}}{A(d_{j,n},f_{i})N(f_{i})B_{s}}\right]=R_{0}$$

Substituting the value of $P_{j,n}^{i}$ from Equation (9) into Equation (10) and applying some straightforward algebraic steps, we get:

(11) $$\lambda =\left[\frac{{\text{Part}}_{1}}{{\text{Part}}_{2}}\right](\ln2),\;\text{where}\;{\text{Part}}_{1}=2^{R_{0}/xB_{s}}\;\text{and}\;{\text{Part}}_{2}={\left(\prod_{i=1}^{x}\frac{{\left|\alpha_{j,n}^{i}\right|}^{2}}{A(d_{j,n},f_{i})N(f_{i})}\right)}^{1/x}$$

Solving for the minimum power $P_{j,n}^{m}$, we get:

(12) $$P_{j,n}^{m}=B_{s}\;{\left(\frac{{\text{Part}}_{1}}{{\text{Part}}_{2}}-\frac{{\text{Part}}_{3}}{{\text{Part}}_{4}}\right)}^{+}\;\text{where}\;{\text{Part}}_{3}=A(d_{j,n},f_{m})N(f_{m})\;\text{and}\;{\text{Part}}_{4}={\left|\alpha_{j,n}^{m}\right|}^{2}$$

The number of subcarriers, *x*, that is used by node *j* to transmit data to node *n*, is determined according to the mode of operation in use, as we will see in Section 5.

## 5. Adaptive Protocol Detailed Design

The proposed protocol can be configured to suit the operational objective for which the network has been established. There are three modes of operation that can be configured; namely, the *Random Mode (RM)*, the *Equal Opportunity Mode (EOM)*, and the *Energy Conscious Mode (ECM)*. We first discuss the network arrangement that we assume in this study. We then describe the three modes of operation of the protocol in details.

### 5.1. OFDMA-MAC Core Module

The OFDMA-based MAC protocol that we propose is built on the OFDMA technology which divides an available channel into a number of orthogonal sub-channels which are termed “subcarriers”. We utilize this technology to enable simultaneous sessions through subcarrier sharing among communicating nodes. Each neighboring pair of nodes uses a subcarrier or a set of subcarriers for data transmission. This used set of subcarriers is, therefore, reserved for the use of this communicating pair until they relinquish it explicitly. The time line is divided into slots each of length T_s_ where transmissions start only at the beginning of each time slot. Synchronization is assumed to be done through a one-hop transmission from the base station.

The objective is to achieve optimal sharing of the available subcarriers. Optimal here means the best distribution of the available subcarriers among network nodes which results in the minimum transmission power consumption subjective to a minimum required throughput levels. Protocol initialization is done as follows:

1. On network initialization, each node sends a short pilot message broadcast to its neighbors over the whole OFDMA subcarrier spectrum. To avoid collisions, each node observes a random backoff period, T_b_off_, before sending its pilot signals. After the backoff period, the node senses the channel. If all subcarriers are free, it sends its pilot message; otherwise it observes another random backoff period. The pilot message may be lost over some subcarriers; however, if at least one copy successfully arrives over any subcarrier, the receiving node, sends back a *Pilot Message Resend Request* to inform the sender that his previous pilot message was not successfully received. The sender resends the pilot message again and waits to receive either a successful reply (see step 2 below) or keep resending the pilot message for a predetermined number of times (PM_r_), after which the sender gives up. It should be noted that this protocol targets only static underwater sensor deployments. This requirement is necessary to reduce the number of pilot messages used to discover neighboring nodes and to build the SNR values over the various OFDMA subcarriers during the initialization phase. When nodes are static, the neighborhood membership is fixed and pilot messages' resend operations rarely occur. On the other hand, mobility increases pilot messages' overhead since a node may have to resend the pilot message PM_r_ times until it discovers that the old neighbor was no longer available due, most probably, to mobility. The pilot message overhead increases energy expenditure if each pilot message is sent many times, as well as, it considerably increases the already big delay that acoustic channels suffer.
2. Upon successfully receiving the pilot messages, each node sorts the signal to noise ratio (SNR) values of the received messages over each subcarrier in a descending order, for each of its neighbors. It then sends a reply to each neighbor informing it of the sorted SNR values of the pilot messages that it has received from this neighbor.
3. After receiving the SNR values from all neighbors, each node selects a common best carrier to be used as a simplex control subcarrier from the node to all of its neighbors. Each node informs its neighbors of its choice of the control subcarrier. Note that the control subcarrier from each node to its neighbors must be different from that of each of the neighbors to avoid contention of control traffic.
4. A node that wants to communicate with a neighbor picks a set of one or more subcarriers in such a way that minimizes its transmission power while providing the required transmission rate, see Section 4. The maximum number of subcarriers that a pair of nodes, S and R, is allowed to use is SC_max_(S 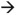
R). If the total number of subcarriers is SC and the number of neighbors of any node *v* is N_b_(*v*), then SC_max_(S 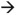
R) is given by:
  (13) $$SC_{\max}(S\to R)=\frac{SC}{\max\left(N_{b}(S),N_{b}(R)\right)}$$

### 5.2. Hidden and Exposed Terminals

To prevent hidden and exposed terminal problem, the sender (S) and the receiver (R) proceed as:

1. Node S picks the set of subcarriers (among the available subcarriers) that optimizes its power transmission, according to the model that we presented in Section 4. It then broadcasts a *Reserve* message of its selected subcarriers over its control subcarrier to all neighbors.
2. If any other neighbor of R has already reserved this set of subcarriers, or part of it, for sending, node R notifies S to exclude the reserved subcarriers.
3. If any other neighbor of S, say B, has already reserved this set of subcarriers, or part of it, for receiving, node B notifies S to exclude the reserved subcarriers.
4. If S receives carrier exclusion feedback from one or more of its neighbors, it selects another best set out of the remaining subcarriers and goes back to step (1). Otherwise, S sends a *Confirm* message of its selected subcarriers over its control subcarrier to all neighbors.
5. When S 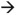
R communication ends, both S and R send a relinquish control message of the used subcarriers over their control subcarriers to all neighbors.

### 5.3. Modes of Operation

If more than one node in the same neighborhood initiates communications at the same time, there may be an overlap on the subcarriers selected by each node. The main idea is to avoid conflict among neighboring nodes by collaboratively agreeing on a subcarrier scheduling. When a node R receives reserve control messages from more than one neighbor at the same time, R initializes a list populated by the requesting nodes called LN and a list of the currently available subcarriers called LS. We use RS(U) to represent the list of requested subcarriers by U and AS(U) to represent the list of the actually assigned subcarriers to U. In the following, we present three different options (modes of operation) for R to assign subcarriers among the possibly conflicting subcarriers' requests. The main concern in the first mode of operation dubbed as Random (RM) Mode (RM) is the simplicity and the speediness of subcarrier assignment. In the second mode of operation dubbed as Equal Opportunity (EOP) Mode, the main concern is to achieve fairness in the subcarrier allocation process among the contending nodes. Finally, the main concern of the last mode of operation dubbed as Energy Conscious (ECM) Mode is to prolong the life time of the network.

#### 5.3.1. Random Mode (RM)

In this mode of operation, a node, say R, randomly assigns subcarriers without paying attention to fairness or network life time. Therefore, the main concern here is to speed up the process of subcarrier allocation and to make it simpler by minimizing the number of control messages' exchanged among the neighboring nodes. Table 1 presents the algorithm that a node, say R, performs as part of the RM subcarrier scheduling process within its neighborhood.

#### 5.3.2. Equal Opportunity Mode (EOM)

In this mode of operation, node R assigns subcarriers fairly among the requesting neighbors. If more than one node requests the same set of subcarriers, each contending node picks a subcarrier and leaves the others to pick one each and the cycle gets repeated. As of which node picks first, we can use attributes or deciding factors such as the absolute energy level of each node (the one with lower energy gets to pick first and so on). Other attributes include node ID and so on. Table 2 presents the algorithm that a node, say R, performs as part of the EOM subcarrier scheduling process within its neighborhood.

#### 5.3.3. Energy Conscious Mode (ECM)

In this mode of operation subcarriers are assigned to the contending nodes on the basis of their available residual energy (RE). The objective here is to prolong the network lifetime by balancing the energy consumption over all the nodes in the network. The network life time here is measured by the time until a certain predefined percentage of nodes in the network deplete their batteries. We assume that all network nodes are honest, *i.e.*, no selfish or malicious nodes exist within the setup. The ECM mode gives the node with the lowest residual energy the right to select the set of subcarriers that best minimizes its energy consumption first, then the node with the next lowest residual energy and so on. Table 3 presents the algorithm that a node R performs as part of the ECM subcarrier scheduling process within its neighborhood,

If a node, say S, needs to initiate a communication session while other nodes (A and B) are currently using part of the subcarriers channel set: Assume the residual energy values in the three nodes are RE(A) < RE(S) < RE(B). Node S computes its best subcarriers using the free subcarriers and the subcarriers used by all the nodes with higher residual energy. Any subcarrier that node S selects and being used by nodes with higher residual energy (node B, in this case) has to be relinquished. This will be done only at the beginning of the next time slot. Nodes that relinquish subcarriers in favor of higher priority nodes will reselect the subcarriers that minimize their energy consumption from the free subcarriers by following the ECM algorithm. However, if the node that has to relinquish subcarriers finds that it will be no longer able to sustain its transmission rate for the current session, it decides not to relinquish its subcarriers and continues to hold on to them until the end of its current transmission session.

## 6. Experimental Evaluation

**Figure 2.**
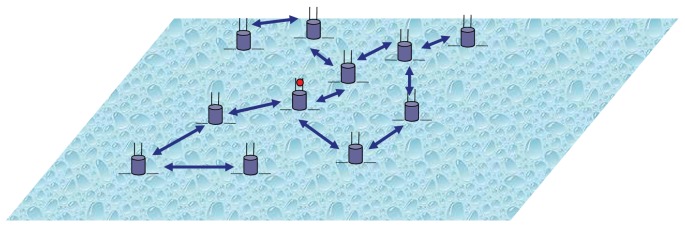
2-D network arrangement.

In order to evaluate the proposed protocol, we conducted several experiments under different operating conditions using the network topology shown in Figure 2. The figure shows a flat (2D) deployment of 100 regular UAWSN nodes uniformly distributed over a field of size 500 × 500 square meters. One sink node (the node with red dot in Figure 2) is positioned at the center of the field and is used to collect data from the regular nodes. A blue two-headed arrow between any two nodes in Figure 2 indicates an existing direct bi-directional communication link between the two ends, while a missing link indicates the absence of direct communication link between the two ends. We use an in-house Java-based simulator that captures the underwater acoustic system and channel models formulated in Sections 3 and 4. In our experiments, we compare the energy cost and the throughput of the new protocol to that of a multi-access protocol. The simulation timeline is divided into equal epochs with transmissions start at the beginning of each time epoch and may continue over parts of the epoch or over several epochs. Each node is initially equipped with the same amount of energy (10 units), and energy consumption results from data transmission. The transmission energy is computed from the transmission rate (a parameter randomly selected during the simulation), the transmission duration and the transmission power as computed from Equation (12). We compare the performance of our protocol with a CDMA-based approach in [21]. We assume that all the nodes are static over the whole simulation time. Unless otherwise indicated for a certain experiment, the simulation parameters that we use are as follows. The channel bandwidth is 24 KHz. The average data rate per session is 2 Kbps. The wireless acoustic sub-channels between two communicating nodes are assumed to undergo a Rayleigh flat fading channel. The total number of OFDMA subcarriers is 256 and the minimum number of the subcarriers to pick should be 4. We run each experiment for at least 1,000 times and present the results with 95% confidence interval. Table 4 summarizes the input parameters that we used in our simulation. The out metrics that we have used are: (i) the energy consumed per transmitted bit averaged over all transmissions by all the nodes over the simulation time; and (ii) the total throughput computed by averaging all the successfully received bits by all the nodes over the simulation time.

### 6.1. Changing Transmission Rate

**Figure 3.**
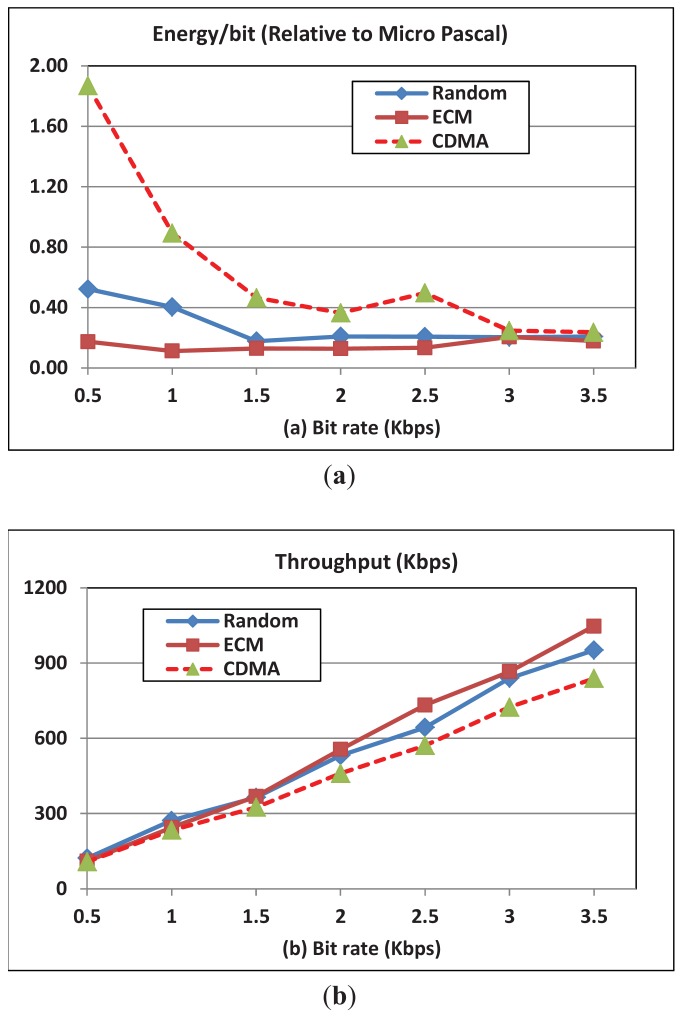
(**a**) Energy per transmitted bit; (**b**) Throughput for OFDMA and CDMA with data rate. BW = 24 KHz; N = 100; Number of subcarriers = 256.

In this experiment, we change the data rate per session and observe throughput and energy consumption per transmitted bit for OFDMA and CDMA. Figure 3(a) presents a comparison of the energy consumption per bit between the CDMA-based techniques and our proposed OFDMA technique with various modes of operation. The results for ECM and EOM are similar; therefore, we only present that of ECM mode. This similarity is a result of the strategy used for carrier assignment in both modes and the assumption of uniform distribution of nodes over the experiment field. ECM prefers nodes with less energy and in EOM distributes carriers in a way that makes energy consumption almost equal on average. Thus, in both modes each node will be fairly assigned carriers relative to the neighboring nodes, which in turn results in an overall similar average performance across all the nodes when computed at the end of the simulation. However, the performance will be different in the two modes (ECM and EOM) if each node is considered separately, which explains the longer network lifetime under ECM compared to EOM as shown in Figure 7. The figure shows that the energy consumption of OFDMA-based protocols is lower than that of the CDMA-based protocols due to the fine control and scheduling of the channel. This is because the bandwidth in CDMA is allocated as one channel while in OFDMA the channel is divided into many independent channels. This channel diversity in OFDMA increases the chances for two communicating nodes to find good channels. On the other hand, a bad channel in CDMA has no substitution, which makes the energy expenditure to increase and the throughput to decrease considerably. Moreover, the channel diversity in OFDMA helps to decrease the interference in dense networks due to the multiple independent channels while in CDMA fewer nodes could communicate due to the possible interference using the single CDMA channel.

The figure also shows that the per bit transmission energy consumption decreases when the transmission rate increases. The absolute energy increases with increasing the transmission rate, however, the normalized energy per bit decreases since the increase in the power required to achieve the requested transmission data rate is much smaller than the increase in the requested data rate. Finally, the figure shows that the per bit energy consumption for random mode (RM) is slightly higher than that of the ECM (and EOM). In the RM mode the decision is made locally and lacks coordination with neighbors, however, in ECM and EOM modes, the decisions are made collectively to optimize the overall energy consumption. Figure 3(b) shows that throughput achieved by our OFDMA-based MAC is higher than that of the CDMA-based MAC mechanisms. Additionally, the throughput in ECM (and EOM) is higher than that of the RM mode. Finally note that, intuitively, the throughput increases with increasing the transmission rate.

### 6.2. Changing Number of Network Nodes (N)

**Figure 4.**
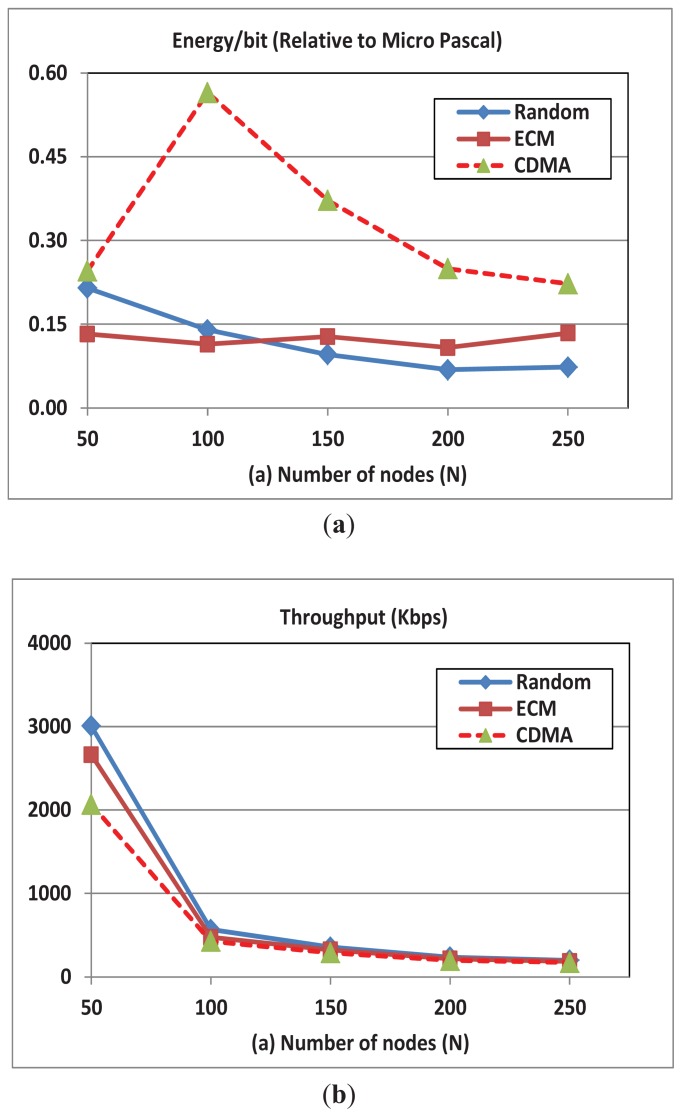
(**a**) Energy per transmitted bit; (**b**) Throughput for OFDMA and CDMA with the change of the number of nodes. BW = 24 KHz; Transmission rate = 2 Kbps; Number of subcarriers = 256.

In this experiment, we change the number of network nodes and measure the resulting effect on throughput and energy consumption. Figure 4(a) shows that with increasing the number of nodes (for bandwidth of 24 KHz and rate of 2 Kbps) the energy cost per bit is stable in the OFDMA-ECM mode, which clearly indicates the scalability of the scheme. Additionally, we note that the energy cost per bit in the OFDMA-Random mode slightly decreases when we increase the number of nodes. However, in CDMA, the per bit energy overhead initially increases when we increase the number of nodes and then starts to decrease (for N ≥ 100). With the increase in the number of nodes, two opposing factors affect the energy cost. On one hand, multi-user diversity improves the possibility of finding good channel conditions as the number of nodes increases thus enhancing the chance to send more data for the same power expenditure. On the other hand, increasing the number of nodes increases the chances of interference (Equation (13)), in case of CDMA, and lowers the number of available subcarriers per node, in case of OFDMA. The two factors balance each other in the case of OFDMA-ECM mode and thus we see the stable behavior. In the case of OFDMA-Random mode the earlier factor always dominates the later and thus we see the continuous decrease in the per bit energy overhead with the increase in N. However, in CDMA the later factor dominates the earlier for small number of nodes (N ≤ 100) and thus we see the initial increase in the per bit energy overhead when the number of nodes increases. When the number of nodes increases beyond appoint (N ≥ 100) the earlier dominates the later and thus we see a decrease in the per bit energy overhead in CDMA. Finally note that the per bit energy consumption in OFDMA is lower than that of CDMA due the more efficient and finer control of the channel.

Figure 4(b) shows that the average throughput in all the schemes decreases with increasing the number of nodes due the increase in the interference and contention for the available channels. The Signal to Noise Ratio formula (SNR) in CDMA is:

(13) $$\text{SNR}\left(d_{j,n},f\right)=\frac{G_{j,j}{\left|\alpha_{j,n}\right|}^{2}P_{j,n}/A(d_{j,n},f)}{N(f)B+\overset{N}{\underset{k\neq j}{\text{∑}}}G_{j,k}{\left|\alpha_{j,n}\right|}^{2}P_{k,n}/A(d_{k,n},f)}$$

where *G_j,k_* captures the spreading gain and/or cross correlation between codes in CDMA. In our experimental simulations we set *G_j,j_*, and *G_j,k_* is modeled as a uniform random variable on the set [0,1], *i.e., G_j,k_*∼U [0,1].

### 6.3. Changing Bandwidth (BW)

**Figure 5.**
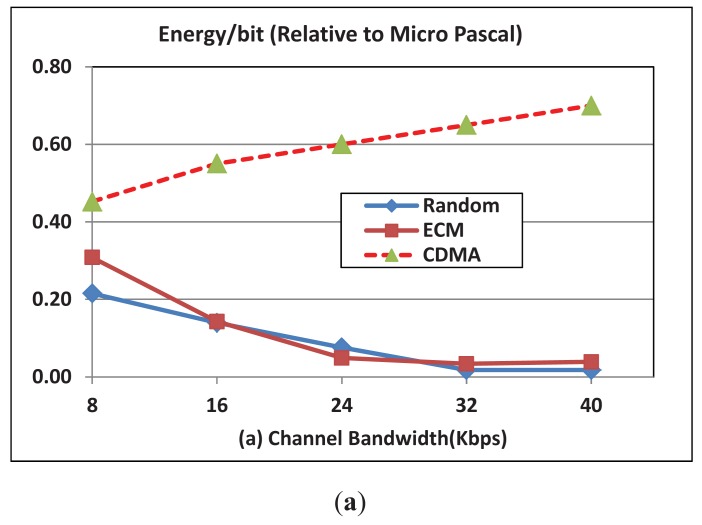

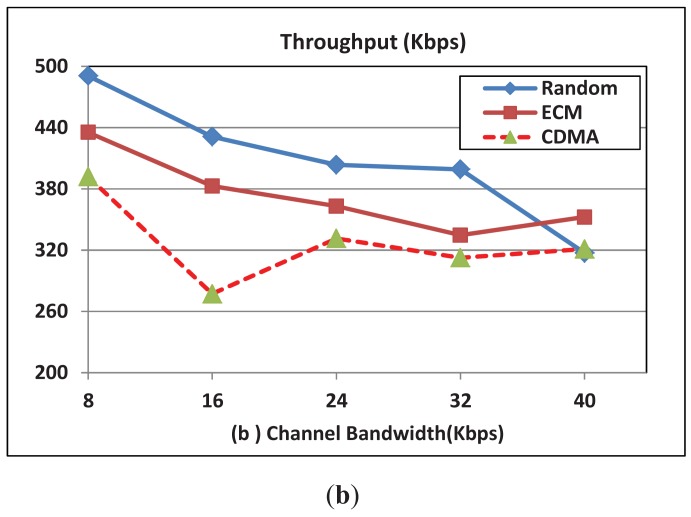
(**a**) Energy per transmitted bit; (**b**) Throughput for OFDMA and CDMA with the change of bandwidth. Number of subcarriers = 256; N = 100; transmission rate = 2 Kbps.

Figure 5(a) shows the effect of bandwidth variations on the per bit transmission energy for both OFDMA (we use the EOM mode) and CDMA schemes. The figure clearly shows that the energy expense in the OFDMA scheme is lower than that of the CDMA schemes. Additionally, the figure shows that energy cost per bit for the CDMA-based protocol increases while it decreases in the case of the OFDMA-based protocol. The increase in the bandwidth results in an increase in the noise which in turn results in an increase in the energy expenditure. However, the diversity of the sub-channels in the OFDMA and the possible simultaneous scheduling of these sub-channels overcome the increase in the noise which in turn results in decreasing the energy expenditure when the BW increases in the OFDMA scheme. Figure 5(b) shows that the throughput in the OFDMA scheme is higher than that of the CDMA schemes. Additionally, the figure shows that the throughput decreases with the BW due to the increase in the noise which decreases the channel reliability and hence the throughput.

### 6.4. Changing the Number of Subcarriers

We examine here the variations in energy consumption and throughput with the number of channel subcarriers.

**Figure 6.**
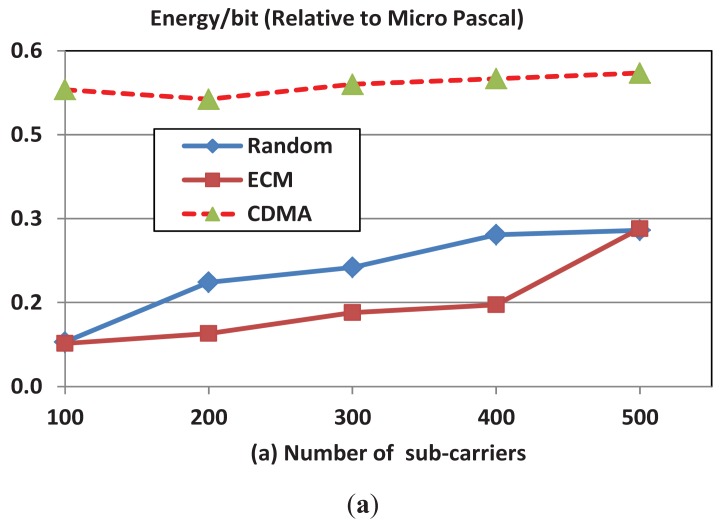

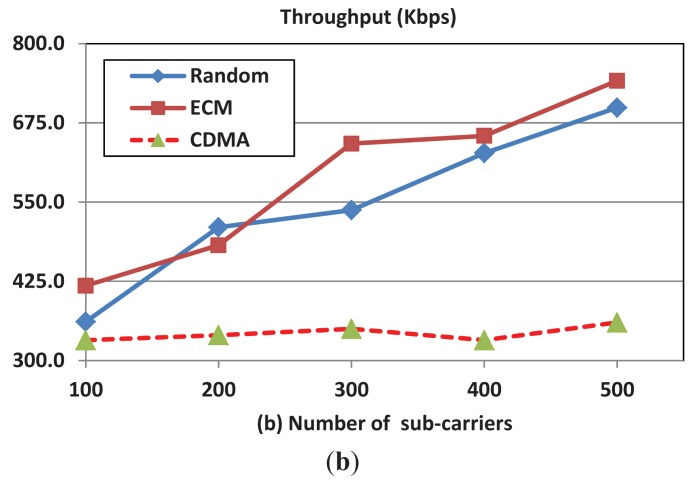
(**a**) Energy per transmitted bit; (**b**) Throughput for OFDMA and CDMA with number of subcarriers. BW = 24 KHz; N = 100; transmission rate = 2 Kbps.

Figure 6(a) shows that the per bit energy consumption of OFDMA scheme increases when the number of subcarriers increases. As the number of subcarriers increases the subcarrier bandwidth decreases and the interference increases as a result of the increase in the number of sending nodes. Therefore, the channel reliability decreases and hence the per bit energy efficiency decreases. Figure 6(b) shows that the throughput increases with the number of subcarriers due to the increase in the number of possible simultaneous sessions when the number of available subcarriers increases. Note that, intuitively, the performance of the CDMA protocol is consistent and is not affected by of the number of subcarriers and we show it here for readers' convenience.

### 6.5. Network Lifetime

**Figure 7.**
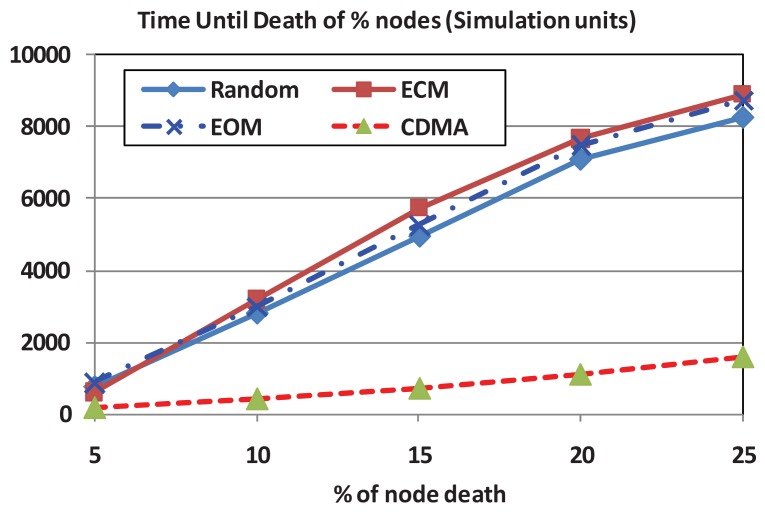
Average network lifetime for both OFDMA and CDMA. BW = 24 KHz; N = 100; transmission rate = 2 Kbps; Number of subcarriers = 256.

In this experiment, we measure the percentage of surviving network nodes with the passage of time. This gives an indicator of the network lifetime which includes both the data transmission energy and the overhead energy. We compare all the three modes of operation of our proposed OFDMA scheme and the CDMA scheme. Figure 7 clearly shows that the network lifetime of all the modes of operation of OFDMA scheme is higher than that of CDMA. For example, for 20% death rate the OFDMA-ECM network life time is seven-fold the network lifetime under the CDMA scheme. This clearly shows that the energy conservation strategy that is followed by the proposed OFDMA scheme could result in extending the lifetime of network nodes (for the simulated scenario) by seven-fold over that of the CDMA-based scheme. The results also show that the OFDMA-ECM mode of operation is the best in terms of network lifetime since its subcarrier scheduling mechanism gives priority to nodes with lower residual energy. This helps to maintain balanced residual energy in each node and therefore, elongate the network lifetime. However, the overhead energy of the ECM is slightly higher than both the RM and the EOM since it requires feedback messages from the nodes about their residual energy levels.

## 7. Conclusions

OFDMA offers multi-user capability which can be exploited to provide interference-free channel sharing in resource constrained underwater acoustic wireless sensor network environments. We propose a MAC protocol utilizing the OFDMA multiple access signaling scheme which is adaptable based on the operational conditions and goals. It consists of three modes of operation; namely, the random mode, the equal opportunity mode, and the energy conscious mode. We derived the model that is used for carrier selection on the basis on optimizing transmission energy. We described the different modes of operation and their objectives. While the equal opportunity mode treats all network nodes as equal within the current transmission time slot (*i.e.*, stateless mode), the energy conscious mode takes into consideration the energy state of network nodes. The random mode is the simplest mode that randomly assigns the available subcarrier. We conducted several simulation experiments to validate the model and the associated protocol against a CDMA-based technique. The results showed that the proposed technique consumes considerably less per bit energy while providing a considerably longer network lifetime over the CDMA-based technique.

As a future extension to this work, we plan to investigate the possible adaptation of the protocol in mobile UAWSN scenarios such us floating ocean deployments or river deployments. Additionally, we plan to evaluate the performance of the proposed protocol under other fading models such as Nakagami-m and Rician. Moreover, even though this work has involved extensive simulation experiments as well as rigorous mathematical analysis to validate the protocol that has been presented, we or others could in the future build a test-bed to provide an even more realistic validation of the work. Finally, we plan to provide comprehensive performance comparison of our protocol against other newly proposed OFDMA-based MAC protocols.

## Figures and Tables

**Table 1.** Pseudo code for the random mode (RM) of operation.

*WHILE* (LN is not empty) *DO*
U ← randomly pick a node from LN
Assign to U the available requested subcarriers
LN ← LN – U
AS(U) ← the available RS(U)
LS ← LS – the available RS(U)
*END*

**Table 2.** Pseudo code for the equal opportunity mode (EOM) of operation.

*WHILE* (LN is not empty) *DO*
U ← pick the node at the head of LN
SC← pick the first subcarrier in RS(U)
AS(U) ← {AS(U) + SC}
RS(U) ← {RS(U) – SC}
IF (RS(U) is empty) THEN
LN ← LN – U
END
IF (SC is requested by another node)
Put U at the tail of LN
*END*
*END*

**Table 3.** Pseudo code for the energy conscious mode (ECM) of operation.

SORT LN with least RE comes at the head
*WHILE* (LN is not empty) *DO*
U ← pick the node at the head of LN
Assign to U the available requested subcarriers
LN ← LN – U
AS(U) ← the available RS(U)
LS ← LS – the available RS(U)
*END*

**Table 4.** Input parameters used in the simulation.

**Parameter**	**Value**	**Parameter**	**Value**

Initial node energy	10 units	Bandwidth	24 KHz
# of OFDMA subcarriers	256	k in Equation (1)	1.5
Simulation time	1,000 epochs	w in Equation (3)	0 m/s
Data rate	2 Kbps	s in Equation (3)	0.5
Field area	500 × 500 m^2^	Number of nodes	100
